# Virus-associated hemophagocytic syndrome as a major contributor to death in patients with 2009 influenza A (H1N1) infection

**DOI:** 10.1186/cc10073

**Published:** 2011-03-02

**Authors:** Gernot Beutel, Olaf Wiesner, Matthias Eder, Carsten Hafer, Andrea S Schneider, Jan T Kielstein, Christian Kühn, Albert Heim, Tina Ganzenmüller, Hans-Heinrich Kreipe, Axel Haverich, Andreas Tecklenburg, Arnold Ganser, Tobias Welte, Marius M Hoeper

**Affiliations:** 1 Departments of Hematology, Hemostasis, Oncology, and Stem Cell Transplantation, Hannover Medical School, Carl-Neuberg-Strasse 1, D-30625 Hannover, Germany; 2 Department of Respiratory Medicine, Hannover Medical School, Carl-Neuberg-Strasse 1, D-30625 Hannover, Germany; 3 Department of Nephrology, Hannover Medical School, Carl-Neuberg-Strasse 1, D-30625 Hannover, Germany; 4 Department of Gastroenterology, Hepatology and Endocrinology, Hannover Medical School, Carl-Neuberg-Strasse 1, D-30625 Hannover, Germany; 5 Department of Cardio-, Thoracic-, Transplantation and Vascular Surgery, Hannover Medical School, Carl-Neuberg-Strasse 1, D-30625 Hannover, Germany; 6 Institute for Virology, Hannover Medical School, Carl-Neuberg-Strasse 1, D-30625 Hannover, Germany; 7 Institute for Pathology, Hannover Medical School, Carl-Neuberg-Strasse 1, D-30625 Hannover, Germany; 8 Hospital Administration, Hannover Medical School, Carl-Neuberg-Strasse 1, D-30625 Hannover, Germany

## Abstract

**Introduction:**

Virus-associated hemophagocytic syndrome (VAHS) is a severe complication of various viral infections often resulting in multiorgan failure and death. The purpose of this study was to describe baseline characteristics, development of VAHS, related treatments and associated mortality rate of consecutive critically ill patients with confirmed 2009 influenza A (H1N1) infection and respiratory failure.

**Methods:**

We conducted a prospective observational study of 25 critically ill patients with 2009 influenza A (H1N1) infection at a single-center intensive care unit in Germany between 5 October 2009 and 4 January 2010. Demographic data, comorbidities, diagnosis of VAHS, illness progression, treatments and survival data were collected. The primary outcome measure was the development of VAHS and related mortality. Secondary outcome variables included duration of mechanical ventilation, support of extracorporeal membrane oxygenation and duration of viral shedding.

**Results:**

VAHS developed in 9 (36%) of 25 critically ill patients with confirmed 2009 influenza A (H1N1) infection, and 8 (89%) of them died. In contrast, the mortality rate in the remaining 16 patients without VAHS was 25% (*P *= 0.004 for the survival difference in patients with or without VAHS by log-rank analysis). The patients were relatively young (median age, 45 years; interquartile range (IQR), 35 to 56 years of age); however, 18 patients (72%) presented with one or more risk factors for a severe course of illness. All 25 patients received mechanical ventilation for severe acute respiratory distress syndrome and refractory hypoxemia, with a median duration of mechanical ventilation of 19 days (IQR, 13 to 26 days). An additional 17 patients (68%) required extracorporeal membrane oxygenation for a median of 10 days (IQR, 6 to 19 days).

**Conclusions:**

The findings of this study raise the possibility that VAHS may be a frequent complication of severe 2009 influenza A (H1N1) infection and represents an important contributor to multiorgan failure and death.

## Introduction

In the spring of 2009, novel human influenza A (H1N1) (A/H1N1/2009) infection began spreading from Mexico around the globe, causing a worldwide pandemic [1-3]. Contrary to initial fears, most patients experienced a mild clinical course. Some patients did become critically ill with respiratory failure, however, requiring intensive care and ventilator support. Mortality rates were high in these patients, especially in those who developed multiorgan failure [4-6].

The mechanisms leading to multiorgan failure and death in patients with influenza infection are not well understood. Septicemia is a leading cause of seasonal influenza, mainly due to secondary infection by other microorganisms, principally Gram-positive or Gram-negative bacteria. The first reports of fatal A/H1N1/2009 infections, however, only described septicemia occasionally [7,8]. Other pathomechanisms may also contribute to severe multiorgan failure, with several reports suggesting that patients with severe influenza infection may develop a virus-associated hemophagocytic syndrome (VAHS) [8-10].

VAHS may present as an aggressive, life-threatening disease, with previous reports implicating its role in fatal cases of seasonal (H3N2) influenza as well as avian (H5N1) influenza virus [8,9,11]. Analogously to hereditary hemophagocytic lymphohistiocytosis (HLH), VAHS is associated with massive cytokine release ("cytokine storm"), elevated plasma levels of soluble interleukin 2 receptor (sIL-2R) and other inflammatory mediators and the accumulation of activated T-lymphocytes and macrophages in various organs, frequently resulting in multiorgan failure and death [12-16].

In Germany, peak infection rates for A/H1N1/2009 occurred between October 2009 and December 2009, that is, in the first winter season after the initial outbreak in Mexico. In our tertiary care center, the first critically ill patient with A/H1N1/2009 infection and respiratory failure was admitted on 5 October 2009. This patient required mechanical ventilation and extracorporeal membrane oxygenation (ECMO) for 2 weeks. The patient's lung function eventually recovered, and the patient was successfully weaned from ECMO support but subsequently died as a result of progressive multiorgan failure 25 days after hospital admission. There was no evidence of secondary septic complications, and VAHS was identified as the most likely cause of multiorgan failure. Therefore, we systematically and prospectively assessed all further patients with A/H1N1/2009 admitted to our intensive care unit (ICU) for the development of VAHS.

This report describes a series of 25 consecutive critically ill patients with severe A/H1N1/2009 infection in whom VAHS was found to be a leading contributor to death.

## Materials and methods

### Study design and patient eligibility

Between 5 October 2009 and 4 January 2010, we prospectively studied 25 adult patients (22 Caucasian, 2 Turkish and 1 Arabian) with confirmed severe A/H1N1/2009 infection admitted to the medical ICU at Hannover Medical School, Hannover, Germany. All critically ill patients were defined as those requiring invasive mechanical ventilation, having a fraction of inspired oxygen level greater than 60% or receiving intravenous infusion of vasopressor or inotropic medication. Additional venovenous ECMO support was necessary in 17 patients. In each case, the diagnosis of A/H1N1/2009 infection was confirmed by real-time reverse transcriptase polymerase chain reaction (RT-PCR) assay [17].

### Data collection

Data collection included patient demographics as well as the presence of the number of predefined comorbidities. Presumed infectious organisms from upper and lower respiratory tract specimens were identified by performing A/H1N1/2009 RT-PCR assays within 48 hours of admission. Further viral, microbiological and fungal surveillance included twice-weekly nasopharyngeal swabs, bronchial lavage, and twice-weekly analysis of blood and urine cultures. In addition to daily routine laboratory analysis, which included C-reactive protein (CRP), procalcitonin, and lactate dehydrogenase (LDH) levels, thrice-weekly measurements of serum ferritin and sIL-2R levels as well as weekly measurements to detect triglyceridemia and hypofibrinogenemia were performed. VAHS was suspected when patients developed fever, cytopenia affecting at least two lineages, hepatitis or splenomegaly and/or when serum levels of sIL-2R and ferritin were increased. The presence of two or more of these features triggered the performance of bone marrow aspiration and biopsy. The diagnosis of VAHS was made according to established HLH diagnostic criteria if three of four major criteria (fever, cytopenia, hepatitis or splenomegaly) and at least one minor criterion (evidence of hemophagocytosis in bone marrow samples or increase in serum level of sIL-2R or ferritin, respectively) were present [18].

We further obtained information regarding the total duration of hospitalization, mechanical ventilation and ECMO support, as well as the duration and use of antiviral, antibiotic and antifungal treatments. Severity of illness was assessed using the Acute Physiology and Chronic Health Evaluation II [19] and Sepsis-related Organ Failure Assessment scores [20]. Severity of illness before the commencement of ECMO was assessed on the basis of ventilatory parameters, arterial blood gas values and chest radiograph findings.

The primary outcome measure was the development of VAHS and VAHS-related mortality. Secondary outcome variables included the duration of mechanical ventilation, ECMO support and the duration of viral shedding.

### Standard treatments

Antiviral treatment consisted of oral oseltamivir at doses of 75 to 150 mg twice daily and/or intravenous zanamivir at a dose of 600 mg twice daily (individually provided on a compassionate use basis by GlaxoSmithKline, Munich, Germany) [21]. The standard therapeutic course for each compound lasted 5 days. If ongoing viral shedding was present, additional treatment courses were administered until A/H1N1/2009 infection was no longer detectable by RT-PCR assay. Early corticosteroid treatment was not routinely used in this patient population.

Patients with VAHS were intended to be treated according to the recommendations of the Histiocyte Society with a modified HLH-94 protocol which consisted of intravenous etoposide (100 to 150 mg/m^2 ^once weekly) and intravenous dexamethasone (8 mg/m^2 ^once daily) [22-24].

Our diagnostic and therapeutic approach was approved by the local institutional review board (Ethics Committee of the Hannover Medical School, Reference 953-2011). In agreement with local regulations, informed consent was waived, as all patients were treated according to the standards of care in our center.

### Statistical analysis

Descriptive analysis was performed using medians and interquartile ranges (IQR). All statistical parameters were tested for normal distribution using the Shapiro-Wilk test of normality. Discrete variables were compared using Pearson's χ^2 ^test or Fisher's exact test. For normally distributed data, continuous variables of patients with and without VAHS were analyzed using the Welch two-sample *t*-test. Otherwise, the Wilcoxon rank-sum test was used. Probability of survival was determined on the basis of survival curves using the Kaplan-Meier method. Differences between groups were calculated using a stratified log-rank test (Fleming-Harrington Gρ family). Hazard ratios for the development of VAHS as a time-dependent variable were evaluated by using a Cox proportional regression model. Last survival status for all patients was assessed on 31 March 2010. Two-sided *P *values <0.05 were considered statistically significant differences. R-Project software version 2.10.1 for Linux was used for statistical computation.

## Results

### Characteristics of patients

Between 5 October 2009 and 4 January 4 2010, 25 adult patients (22 Caucasian, 2 Turkish and 1 Arabian) fulfilled the study's eligibility criteria. All patients were admitted with severe respiratory failure requiring invasive mechanical ventilation (*n *= 25, 100%) and venovenous ECMO support (*n *= 17, 68%). The median age was 45 years (IQR, 35 to 56 years), and 16 patients (64%) were men. Seven of these patients (28%) had no preexisting medical conditions, whereas 18 patients (72%) presented with one or more risk factors, including obesity (*n *= 10), cardiovascular disease (*n *= 8), chronic pulmonary disease (*n *= 4), chronic renal insufficiency (*n *= 4), immunosuppressive therapy after organ transplantation (*n *= 3), diabetes mellitus (*n *= 3), liver disease (*n *= 2), malignant lymphoma (*n *= 2) and pregnancy (*n *= 2) (Table 1). In all patients, A/H1N1/2009 infection was identified by RT-PCR assay, whereas seasonal subtypes of influenza A were not detectable.

**Table 1:** Baseline demographic and clinical characteristics of critically ill patients with H1N1 infection^a^

Characteristics	All patients (*n *= 25)	Patients with VAHS (*n *= 9)	Patients without VAHS (*n *= 16)	*P *value
Median age, yr (IQR)	45 (35 to 56)	53 (39 to 56)	38 (32 to 52)	0.32
Sex, F/M	9/16	2/7	7/9	0.52
Any comorbidity, *n *(%)^b^	18 (72%)	5 (56%)	13 (81%)	0.36
Obesity, *n *(%)^c^	10 (40%)	3 (33%)	7 (44%)	0.67
Median APACHE II score at admission (IQR)	21 (19 to 30)	28 (23 to 32)	21 (18 to 23)	0.29
Median SOFA score at admission (IQR)	11 (10 to 13)	13 (11 to 16)	11 (9 to 12)	0.22
Median duration of mechanical ventilation, days (IQR)	19 (13 to 26)	25 (17 to 26)	18 (11 to 25)	0.69
Patients on ECMO support, *n *(%)	17 (68%)	9 (100%)	8 (50%)	0.02^e^
Median duration of ECMO support, days (IQR)	10 (6 to 19)	10 (4 to 19)	11 (8 to 20)	0.90
Median duration of viral shedding, days (IQR)	19 (14 to 26)	21 (14 to 26)	15 (12 to 22)	0.13
Patients treated with oseltamivir, *n *(%)^d^	24 (96%)	9 (100%)	15 (94%)	0.44
Median duration of oseltamivir treatment, days (IQR)	7 (4 to 10)	10 (5 to 12)	7 (4 to 10)	0.32
Patients treated with intravenous zanamivir, *n *(%)^d^	15 (60%)	6 (67%)	9 (56%)	0.61
Median duration of zanamivir treatment, days (IQR)	7 (5 to 12)	6 (5 to 7)	10 (5 to 13)	0.32
Median peak CRP level, mg/l (IQR)	313 (271 to 344)	337 (324 to 345)	302 (241 to 315)	0.03^f^
Median peak LDH level, U/l (IQR)	1,175 (703 to 3,744)	3,819 (1,096 to 9,403)	933 (674 to 1,729)	0.03^g^
Median peak serum sIL-2R level, kU/l (IQR)	2,289 (1,416 to 5,793)	8,188 (5,120 to 10,650)	1,433 (1,092 to 1,904)	0.001^f^
Median peak serum ferritin level, μg/l (IQR)	1,067 (835 to 5,986)	7,576 (4,708 to 68,070)	861 (487 to 1,060)	<0.001^g^
Patients requiring renal replacement therapy, *n *(%)	14 (56%)	8 (89%)	6 (38%)	0.03^e^
Mortality, *n *(%)	12/25 (48%)	8/9 (89%)	4/16 (25%)	0.004^h^

^a^VAHS, virus-associated hemophagocytic syndrome; IQR, interquartile range; APACHE II, Acute Physiology and Chronic Health Evaluation II; SOFA, ; ECMO, extracorporeal membrane oxygenation; CRP, C-reactive protein; LDH, lactate dehydrogenase; sIL-2R, soluble interleukin-2 receptor; ^b^comorbidities were obesity, cardiovascular or chronic pulmonary disease, chronic renal insufficiency, immunosuppressive therapy after organ transplantation, diabetes mellitus, liver disease, malignant lymphoma and pregnancy (see Materials and methods for details); ^c^obesity was defined as body mass index >30 kg/m^2^; ^d^patients received sequential therapy, that is, antiviral therapy was started with oseltamivir but was switched to intravenous zanamivir in patients with persistent viral shedding; one patient received intravenous zanamivir as initial therapy; ^e^difference between VAHS and non-VAHS was significant based on Fisher's exact test for count data; ^f^difference between VAHS and non-VAHS was significant based on the Welch two-sample *t*-test; ^g^difference between VAHS and non-VAHS was significant based on the Wilcoxon rank-sum test; ^h^difference between VAHS and non-VAHS was significant based on log-rank analysis.

### Severity of illness

The median durations of mechanical ventilation and ECMO support were 19 days (IQR, 3 to 26 days) and 10 days (IQR, 6 to 19 days), respectively. Before ECMO commencement, patients had a median respiratory rate of 24 breaths/minute (IQR, 20 to 26/breaths/minute), a median positive end-expiratory pressure of 18 cmH_2_O (IQR, 15 to 20 cmH_2_O) and a median peak airway pressure of 34 cm H_2_O (IQR, 31 to 36 cm H_2_O). The median partial pressure of oxygen in arterial blood (PaO_2_) level was 66 mmHg (IQR, 56 to 85 mmHg), with a PaO_2_/fraction of inspired oxygen ratio of 85 mmHg (IQR, 59 to 138 mmHg). In the course of critical illness, 21 patients (84%) received vasopressor or inotrope therapy and 14 patients (56%) received renal replacement therapy.

### Antiviral treatment and virus shedding

Oseltamivir was used as antiviral treatment in 24 patients (96%) for a median of 7 days (IQR, 4 to 10 days), and zanamivir was used as antiviral therapy in 15 patients (60%) for a median of 7 days (IQR, 5 to 12 days). The median duration of viral shedding from disease onset to the last positive A/H1N1/2009 infection RT-PCR assay was 19 days (IQR, 14 to 26 days). In patients without VAHS, the median viral shedding time was 15 days (IQR, 12 to 22 days) as opposed to a median of 21 days (IQR, 14 to 26 days) (*P *= 0.13) in patients with VAHS.

### Occurrence of VAHS

**Figure 1:**
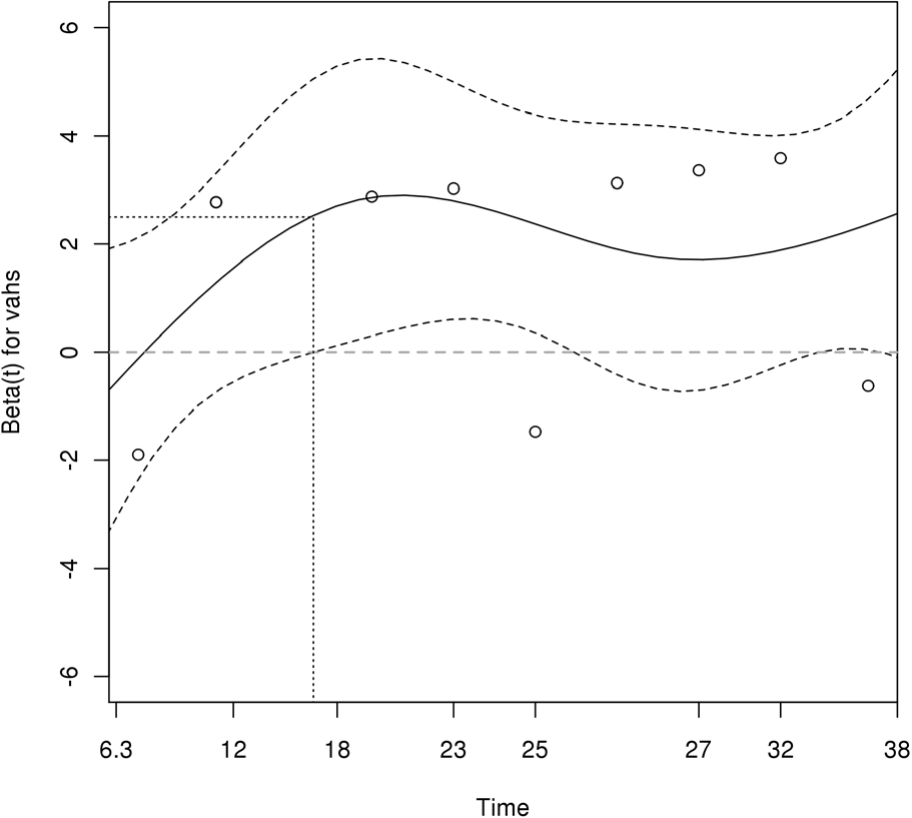
**Predicted hazard ratio for the development of virus-associated hemophagocytic syndrome (VAHS) revealed a 12-fold increase (log-hazard ratio, 2.5) within the first 16 days after symptom onset**.

**Figure 2:**
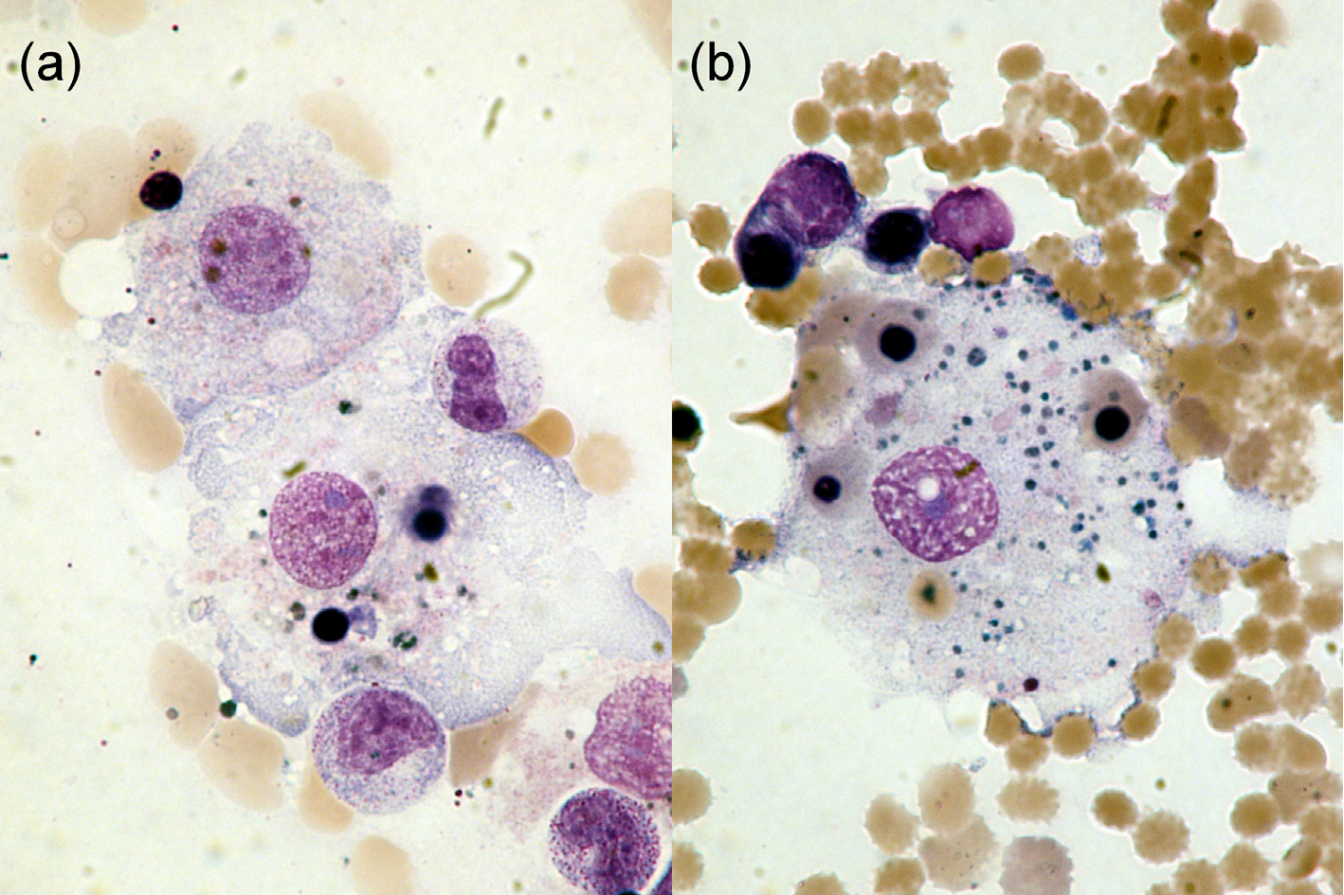
**Bone marrow smears showing large histiocytes with vacuolated cytoplasm phagocytic granulocytes (a) and containing nucleated red blood cells (erythrophagocytosis (b)) (Wright-Giemsa stain; original magnification, ×600)**.

Nine patients (36%) fulfilled the diagnostic criteria for VAHS. The median time from the onset of symptoms to the diagnosis of VAHS was 23 days (IQR, 15 to 29 days), and the median time from admission to the ICU to the diagnosis of VAHS was 16 days (IQR, 11 to 25 days). Within the first 16 days after symptom onset, the predicted hazard ratio revealed a 12-fold increase (log hazard ratio, 2.5) for the development of VAHS (Figure 1). When VAHS was diagnosed, patients demonstrated cytopenia affecting at least two lineages, hepatitis or splenomegaly with a bone marrow specimen demonstrating characteristic features of hemophagocytosis (Figure 2). At the same time, serum analysis revealed markedly elevated levels of ferritin, sIL-2R, LDH and CRP (Table 1). However, there was no evidence of uncontrolled bacterial infection in any of these patients on the basis of repeated sterile cultures from the tracheobronchial tree, blood and urine. Over the course of time, patients who presented with VAHS developed multiorgan dysfunction with hepatitis (*n *= 9, 100%), renal failure (*n *= 8, 89%), pancytopenia (*n *= 8, 89%) and lactic acidosis (*n *= 7, 78%).

### VAHS-directed therapy and mortality

**Figure 3:**
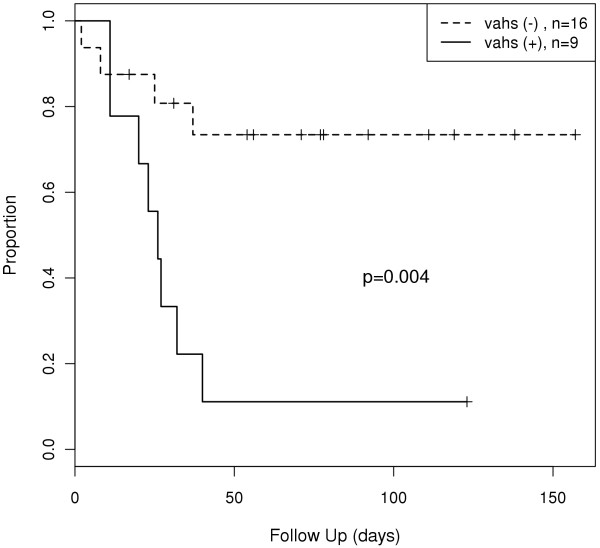
**Kaplan-Meier curve showing estimated survival rates of patients with 2009 influenza A (H1N1) infection with or without virus-associated hemophagocytic syndrome (VAHS)**. *P *= 0.004 by log-rank analysis.

Treatment of VAHS was started in six of the nine patients with VAHS (*n *= 4 with etoposide and dexamethasone and *n *= 2 with steroids only). Three patients were moribund when VAHS was diagnosed and were no longer considered candidates for treatment with etoposide and dexamethasone. Despite VAHS-directed therapy, five of the six patients who were treated died as a result of uncontrolled disease progress leading to multiorgan failure. Overall, eight (89%) of the nine patients with confirmed VAHS died compared to 4 (25%) of 16 patients without VAHS (Figure 3). This difference was statistically significant (*P *= 0.004). Overall, 12 patients (48%) died, all as a result of multiorgan failure.

## Discussion

The present case series confirms previous postmortem analyses that A/H1N1/2009 infection can cause severe and fatal infections in humans, even in the absence of risk factors [25]. More importantly, our data show that VAHS should be taken into consideration as a major pathogenetic mechanism contributing to multiorgan failure and death in patients with severe viral infections.

### Summary of study findings

The occurrence of VAHS in approximately one-third (9 of 25, 36%) of our patients was unexpected. Of the nine patients diagnosed with VAHS, eight (89%) failed to survive. By comparison, only 4 (25%) of the remaining 16 patients without VAHS died, suggesting that VAHS development either contributes greatly to or is itself causative of death in this patient population. On the basis of our initial experience, we prospectively screened all patients admitted to our ICU with A/H1N1/2009 infection for the development of VAHS, and it is therefore unlikely that we missed cases. VAHS was not an initial feature of A/H1N1/2009 infection but developed a median of 23 days (IQR, 15 to 29 days) after symptom onset and a median of 16 days (IQR, 11 to 25 days) after ICU admission. The duration of viral shedding tended to be longer in patients with VAHS than in those who did not develop VAHS. Although this difference is not statistically significant, it supports our hypothesis that prolonged clearance of influenza A (A/H1N1/2009) virus infection may lead to the development of an initial pulmonary hemophagocytosis followed by secondary systemic manifestation. Notably, in all patients who developed VAHS, A/H1N1/2009 infection was still detectable by RT-PCR assay when the syndrome was diagnosed, suggesting that persistent A/H1N1/2009 infection might have been a trigger of VAHS in our patient population.

### Study strengths and limitations

To date, VAHS has mostly been reported in postmortem analyses of patients infected with A/H1N1/2009 [25-27], raising the question whether this syndrome was disproportionately prevalent in our series or whether it was underdiagnosed in others. In the ICU setting, the clinical pattern of VAHS often mimics septicemia, and thus patients with VAHS may easily be misdiagnosed with septic multiorgan failure.

The mortality rate in our series, however, appears higher than those reported in other series of patients with severe A/H1N1/2009 infection [5,28]. In contrast to the practice at some other centers, we did not routinely administer early corticosteroid therapy, as this approach is not supported by robust data [29-32]. We cannot exclude the possibility that our strategy of avoiding corticosteroids in the early phase of A/H1N1/2009 infection may have contributed to the high incidence of VAHS and the rather poor outcomes in our cohort of patients.

The use of ECMO may also have been a risk factor for the development of VAHS. All patients in our series who developed VAHS had received ECMO support for some time during the course of their illness, and eight of nine were still receiving ECMO therapy when VAHS was diagnosed. VAHS did not occur in patients without ECMO support. Although the pathogenesis of VAHS is incompletely understood, there is ample evidence that extensive cytokine activation is a key factor [33]. It is conceivable that the use of ECMO could have been a trigger or an amplifier of cytokine activation [34]. These aspects should be further studied, especially as ECMO has been widely used in patients with severe A/H1N1/2009 infection in ICUs around the globe.

It is generally recommended that patients with VAHS be treated with dexamethasone and etoposide according to a modified HLH-94 protocol, although the efficacy of this regimen is less well established in VAHS than in hereditary hemophagocytic lymphohistiocytosis [22,23,35]. Although antiviral defense might be hampered by the use of dexamethasone or etoposide therapy, there is evidence that early treatment may improve survival in patients with VAHS associated with Epstein-Barr virus infection or influenza A (H5N1) virus infection, respectively [36-38]. The potential mechanism of action is believed to be modulation of the (hyper)activated inflammatory response [9]. In our case series, the development of VAHS was associated with rapid clinical deterioration and the development of multiorgan failure. Treatment of VAHS with etoposide and/or corticosteroids did not prevent a fatal outcome in the majority of our patients. In considering the refractory course exhibited in patients receiving treatment, it remains impossible to determine whether this reflects an overall lack of treatment efficacy, an unknown harmful effect stemming from the treatment or the result of late treatment initiation, that is, when patients had already developed terminal multiorgan failure.

## Conclusions

In summary, our findings raise the possibility that VAHS may be a frequent complication of severe A/H1N1/2009 infection and represents an important contributor to multiorgan failure and death in these patients. Therefore, physicians' awareness and timely diagnosis of VAHS is crucial for early and successful treatment. These observations are preliminary, but may nevertheless have important implications for future management of patients with A/H1N1/2009 infection as well as other severe viral infections.

## Key messages

• Severe A/H1N1/2009 infection is frequently associated with VAHS.

• VAHS represents an important contributor of multiorgan failure and death.

• Physicians' awareness of and regular screening using VAHS diagnostic criteria are crucial for the timely diagnosis of VAHS.

• Early treatment of VAHS with corticosteroids and/or etoposide may improve patient outcomes.

## Abbreviations

APACHE II: Acute Physiology and Chronic Health Evaluation II; CRP: C-reactive protein; ECMO: extracorporeal membrane oxygenation; HLH: hereditary hemophagocytic lymphohistiocytosis; ICU: intensive care unit; IQR: interquartile range; LDH: lactate dehydrogenase; PCT: procalcitonin; RT-PCR: reverse transcriptase polymerase chain reaction; SIL-2R: soluble interleukin 2 receptor; SOFA: Sepsis-related Organ Failure Assessment; VAHS: virus-associated hemophagocytic syndrome.

## Competing interests

The authors declare that they have no competing interests.

## Authors' contributions

GBE, MED, AGA, MHO and JKI designed the research. GBE, MED, TGA, CHA, AHE, MHO, JKI, CKU, HKR, OWI and ASC performed the research. MHO, ATE and TWE contributed new drugs. GBE, TGA and OWI collected data. GBE, MED, AGA, MHO, TGA, CHA, JKI, CKU, ASC and TWE analyzed and interpreted data. GBE performed statistical analysis. GBE, MED, AGA, TGA, CHA, AHE, MHO, JKI, HKR, ASC and TWE wrote and/or critically revised the manuscript. All authors read and approved the final manuscript.
